# Urinary Reagent Strip: A Reliable and Rapid Bedside Diagnostic Tool for Meningitis

**DOI:** 10.7759/cureus.79642

**Published:** 2025-02-25

**Authors:** Nadir Hussain, Rafi U Khan, Asadullah Farooq, Anum Mati

**Affiliations:** 1 Neurology, Hull University Teaching Hospitals NHS Trust, Hull, GBR; 2 Internal Medicine, Ameer-ud-Din Medical College, Lahore, PAK; 3 Medicine, Medical Teaching Institution Bannu, Bannu, PAK

**Keywords:** cerebrospinal fluid, meningitis, remote areas, urinalysis, urinary reagent strip

## Abstract

Introduction: Meningitis, being one of the life-threatening diseases, always needs timely initiation of treatment to save disability and mortality, and this is greatly dependent on early diagnosis. The gold standard way of diagnosing meningitis is to test cerebrospinal fluid (CSF) by microscopy, biochemistry, and cultures, which always require experts and advanced labs, which are not always present in remote areas of underdeveloped countries. This study primarily aimed to assess the accuracy of urinary reagents in diagnosing bacterial meningitis and its comparison with the gold standard method as this could be used for quick analysis of CSF to diagnose meningitis. This could be a very useful tool in resource-limited settings given its rapid accessibility and cost-effectiveness.

Method: This cross-sectional prospective study was conducted at the Department of Neurology, Punjab Institute of Neurosciences (PINS), Lahore, Pakistan, from December 2, 2020, to June 2, 2021. Both genders and ages 14-70 with suspicion of meningitis were included in this study. Biochemistry and microscopy labeled the patients "positive," with a white blood cell count of 10/mm^3^, proteins > 45 mg/dL, and glucose > 40 mg/dL in CSF. Positive urine reagent strips were marked as "positive" if glucose levels were > +1 and protein levels were +2 and if leukocyte esterase levels were positive (qualitatively).

Results: Among the 147 patients, 93 (63%) were males, and 54 (37%) were females. The mean age was 41.82±15.5 years. There were 63 (42.86%) positive results on urine reagent strips and 64 (43.54%) positive results on CSF microscopy and biochemistry. Urine reagent strips had diagnostic accuracy of 92.52%, sensitivity of 90.62%, specificity of 93.98%, positive predictive value (PPV) of 92.06%, and negative predictive value (NPV) of 92.86% with significant p-values (0.001).

Conclusion: CSF pleocytosis could be detected using urine reagent strips, which would be useful in settings without the infrastructure to exclude the diagnosis of bacterial meningitis if negative.

## Introduction

The 2016 Global Burden of Disease (GBD) Study highlights a rise in meningitis instances, with reported cases increasing from 2.50 million in 1990 to 2.82 million in 2016 [1]. Within the United States, the annual incidence of cases stands at 1.33 per 100,000 individuals [2]. Even though there has been a notable 21.0% reduction in meningitis-related mortality from 1990 to 2016, the disease continues to pose a significant burden, especially in developing countries where the healthcare system lacks adequate infrastructure [3]. Inflammation of meninges can be caused by bacterial, fungal, viral, and parasitic organisms, but bacterial meningitis is the most severe and fatal condition. Bacterial meningitis incidence in children under five years old persists as one of the leading causes of mortality within this age demographic, ranking among the top 10 [4,5].

A high morbidity rate is associated with the disease, with about 20% of survivors suffering permanent disabilities, such as neurologic impairments, hearing impairments, and cognitive changes [3]. Hence, it would not be wrong to say that these post-meningitis sequelae have a significant impact on the quality of life, and they put a financial burden on health departments and the families of those affected [6]. This acts as a dual-edged sword, causing an economic burden on the health system while causing significant effects on the patient’s families as they lose their bread-earning hand. Therefore, timely identification and treatment can alleviate the morbidity associated with the disease [7]. The following are signs of bacterial meningitis: cerebrospinal fluid (CSF) appears cloudy, the protein concentration in the CSF rises, the glucose concentration in the CSF drops, and the opening pressure of CSF is elevated; CSF pleocytosis and positive CSF bacterial cultures are indicators of meningitis [8].

The nonspecific nature of the early course of a disease resembling common flu and influenza makes things more challenging, and this can add further difficulties in diagnosing meningitis for already overburdened healthcare professionals in resource-limited settings. A CSF culture is the gold standard for diagnosis in laboratories with adequate facilities [9]. In low-resource settings, laboratory analyses for biochemistry, cytology, and microbiology are often not available, leading to delayed or missed diagnosis and hence delayed treatment, which can result in significant complications [10]. Van de Beek et al., in a forward-looking investigation encompassing 696 instances of bacterial meningitis in adults, discovered that 95% of the individuals exhibited a combination of two among the following four symptoms: fever, headache, stiff neck, and altered mental status [11].

However, only 44% of cases of meningitis showed symptoms of headache, fever, and neck stiffness, which poses a big challenge to rely only on clinical examination findings to diagnose meningitis [12]. In most cases, clinical examination did not reveal focal neurologic deficits, which further complicates this.

Despite the majority of cases with neck stiffness having bacterial meningitis, meningeal signs are insensitive for diagnosis, such as the Kerning sign with 5% sensitivity, the Brudzinski sign with 5% sensitivity, and Nuchal rigidity with 30% sensitivity [13]. These results highlight the constraints of depending on the clinical examination for the diagnosis of meningitis. This is very alarming especially because of the rapid worsening of such patients and often leading to death. Therefore, the lumber puncture should not be delayed because of the absence of meningeal signs as the analysis of CSF is imperative for reaching a diagnosis [8].

In low-resource settings, physicians mostly rely on clinical findings and the physical appearance of the CSF, but these methods have poor diagnostic yields, leading to misdiagnosis and treatment [14]. As a consequence, affordable, rapid, and accurate methods are essential for diagnostic purposes, particularly in health systems with limited laboratory facilities availability.

With all these limitations and constraints, urinary reagent strips provide an alternative method [15]. In low-resource settings without adequate laboratory facilities for standard meningitis diagnostics, we aimed to assess the diagnostic validity of urine reagent strips for detecting bacterial meningitis.

## Materials and methods

The Ethical Review Board of PINS Hospital approved the project. This cross-sectional study was conducted by the Department of Neurology at the Punjab Institute of Neurosciences (PINS), Lahore, Pakistan. The study duration was from 02-12-2020 to 02-06-2021. A non-probability consecutive sampling technique was used.

Patients presented and admitted to the Neurology Department of PINS with suspected meningitis were included in the study with the following inclusion and exclusion criteria:

Inclusion criteria: Both genders aged 14-70 years with suspected meningitis (presence of fever: >100.4 F, headache, vomiting, and stiff neck) were included in the study.

Exclusion criteria: Patients who could not have lumbar puncture (LP) because of radiological evidence of contraindication of LP and local pathology of the spine at the site of LP or and patients who had bleeding diathesis (INR > 1.2) were not included. Patients who refused LP and were not willing to participate were also excluded.

We used Chemstrip 10 MD urine test strips (Roche Diagnostics Corp., Indianapolis, IN) for CSF analysis, keeping laboratory results as the gold standard. After taking a detailed history and performing a thorough clinical examination, the patients underwent lumbar LP. Leukocytes, pH of body fluid, protein, erythrocytes, and glucose were measured using urine reagent strips.

After stratification, diagnostic accuracy was calculated, and a p-value of ≤ 0.05 was considered significant. Mean and standard deviation (descriptive statistics) were calculated for age, while frequencies with percentages were given for qualitative variables such as gender and positive urinary strip test, and a 2x2 table was constructed. Data were stratified for age and gender, and a p-value of equal or less than 0.05 was taken as significant.

## Results

**Figure 1:**
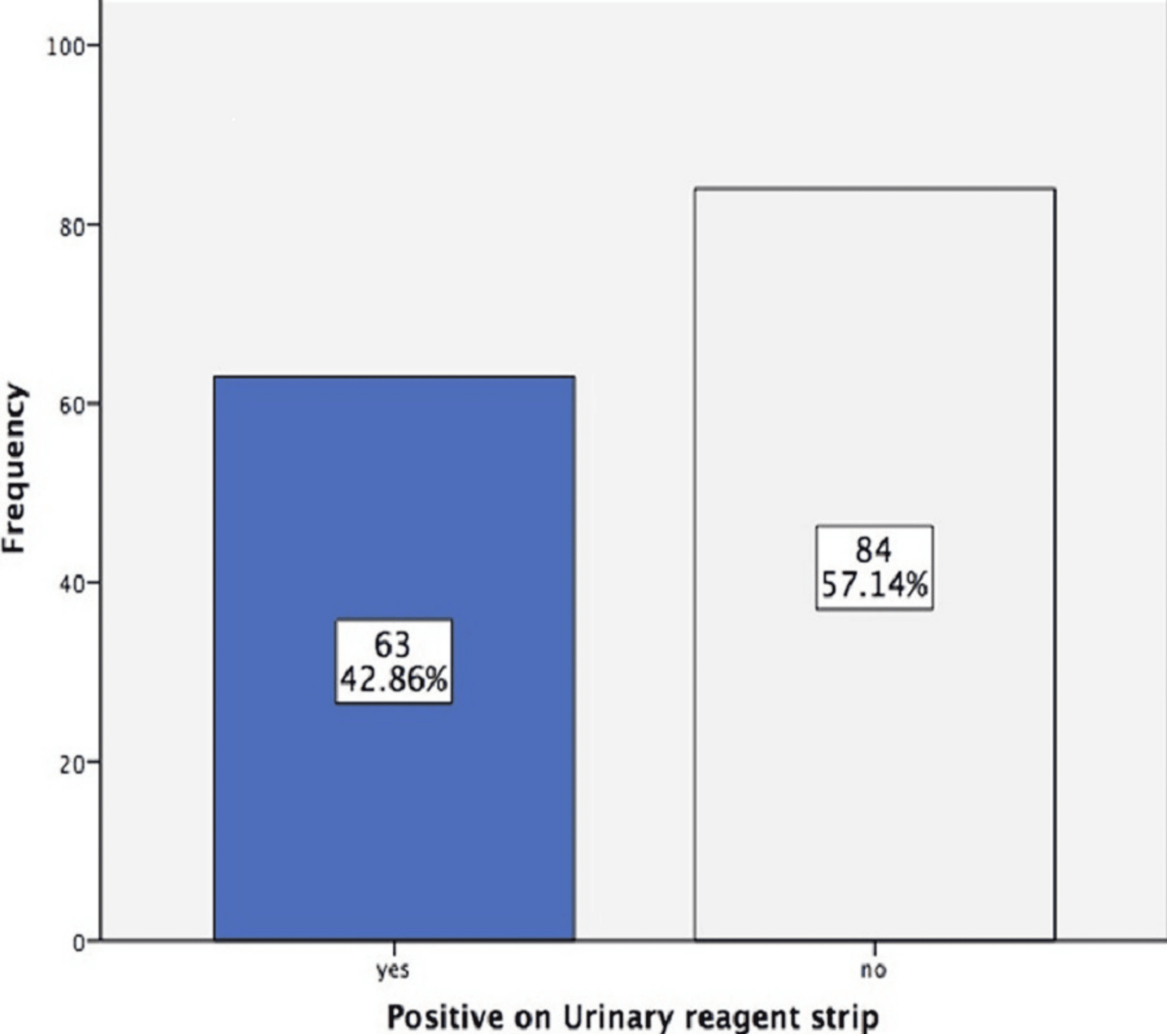
Positive results on the urinary strip reagent (n = 147)

**Figure 2:**
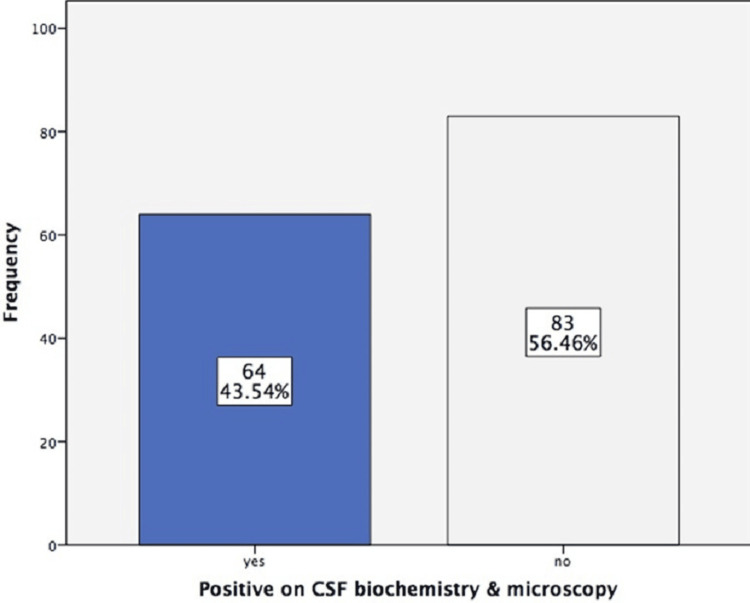
Positive result on cerebrospinal fluid (CSF) microscopy and biochemistry (n = 147)

There were 147 cases in this study, with 93 (63.27%) males and 54 (36.73%) females. The mean age was 41.82±15.54 years. A positive urine reagent strip result was detected in 63 (42.86%) of the cases (Figure 1). The positive CSF microscopy and biochemistry result was detected in 64 (43.54%) out of 147 (Figure 2).

The diagnostic accuracy of the urinary reagent strip was 92.52%, with a sensitivity of 90.62%, specificity of 93.98%, positive predictive value (PPV) of 92.06%, and negative predictive value (NPV) of 92.86%, with p = 0.0001 (Table 1). Diagnostic accuracy was significantly higher in the male gender, with an accuracy of 91.40%. For the female gender, this was 94.44%, with p = 0.001. For the age group 14-39 years, sensitivity was 92.11, with a specificity of 91.18% and diagnostic accuracy of 91.67%, with p = 0.001. For the age group 40-70 years, sensitivity and specificity were 92% and 94%, respectively, while diagnostic accuracy was 93.33% with p = 0.001.

**Table 1:** Diagnostic accuracy of the urinary reagent strip (n = 147) TP: True positive; TN: True negative; FN: False negative; FP: False positive; PPV: Positive predictive value; NPV: Negative predictive value

CSF Microscopy & Biochemistry	Positive on the Urinary Reagent Strip		Negative on the Urinary Reagent Strip		P-value	Sensitivity	Specificity	PPV	NPV	Accuracy
Yes	TP	58	FP	05	0.0001	90.62%	93.98%	92.06%	92.86%	92.52%
No	FN	06	TN	78						

## Discussion

Acute meningitis can pose a life-threatening situation because of the inflammation of the meninges [16]. Several medical tests are required to diagnose meningitis in a timely manner, including CSF microscopy, CSF chemistry, and microbiological tests. Glucose and protein levels need to be measured, CSF cell counts need to be estimated, and these facilities are not available in resource-limited centers [14]. Most centers take six to eight hours to get results for biochemistry tests, eight to 24 hours for Gram stain tests, and 72 hours for cultures. Numerous community hospitals lack the facility of CSF analysis, which is considered to be the backbone of healthcare. Therefore, it is critical for developing countries to have simple diagnostic methods and primary referral centers. The quantification of CSF cellularity and chemistry using urine reagent strips has been carried out; however, the outcomes exhibit variability [17]. This variability among different studies revealing different values for sensitivity and specificity especially the value for glucose indicates that a further structured approach regarding testing protocol is needed.

As per the findings reported by Bhat et al., a positive result demonstrated a high sensitivity of 100% in identifying CSF proteins, exceeding 30 mg/dL, contributing significantly to the diagnosis of meningitis [18]. As indicated by Bortcosh et al., a total of 13 investigations, involving 2,235 subjects, demonstrated that the application of urine reagent strips facilitated the identification of CSF pleocytosis, achieving a sensitivity of 92%, a specificity of 98%, and a negative predictive value of 99% in contexts where bacterial meningitis was prevalent [19]. The significance of this detection remained consistent across both genders based on the findings of the present study. Additionally, in this research, both age groups had significantly better diagnostic accuracy, but similar cut-off points were not applied [19].

Gupta et al. reported that strip tests exhibited a sensitivity of 99% and a specificity of 54% for proteins. Regarding glucose, the strip demonstrated both sensitivity (92%) and specificity (98%) [20]. Notably, the strip displayed high sensitivity (100%) and specificity (96%) for leukocytes at a concentration of 10 cells/cm³. The sensitivity and specificity for CSF erythrocytes were reported as 100%.

In a study by Mazumder et al., urine reagent strips showed a sensitivity of 89.28% and a specificity of 98.61% for leukocytes. Protein concentrations exceeding 30 mg/dL were identified, with a sensitivity of 85.71% and specificity of 95.65% using the reagent strip test. Although reagent strip tests for glucose demonstrated high sensitivity (100%), their specificity was comparatively lower [21].

Romanelli et al. conducted a comparison between the outcomes derived from reagent strips and those obtained through standard cytological and biochemical assays [22]. They provided statistical values for sensitivity, specificity, PPV, and NPV at 90.7%, 98.1%, 95.1%, and 96.4%, respectively, particularly in relation to bacterial meningitis [22].

Kumar et al. identified a significant positive association between the strip measurements and the laboratory results pertaining to the diagnosis of meningitis, with correlation coefficients of K = 0.94, 0.819, and 0.819 for the cellular count, protein concentration, and glucose levels, respectively, and a p-value of less than 0.0001, indicating statistical significance [23].

According to Maclennan et al.'s study, new information can be obtained by using the nitrate patch test, which is also observed as a component of 10-parameter urinary reagent strips, in cases of bacterial meningitis when granulocytes are raised but have not released esterase enzymes [24].

In a comparable context, Joshi et al. utilized reagent strips for comparative analysis, uncovering a sensitivity of 85.2% and a specificity of 89.6% for leukocytes surpassing 10 cells/cumm. Importantly, at a threshold of 30 mg/dL for proteins, the sensitivity was remarkable (98.1%), although the specificity was comparatively modest (57.1%). Conversely, at a threshold of 100 mg/dL, both sensitivity and specificity were regarded as satisfactory. Moreover, in the evaluation of strip outcomes, a threshold of > 40 mg/dL for glucose revealed a markedly elevated specificity (96.5%) relative to sensitivity (61.1%). Similarly, a threshold of 50 mg/dL showcased a significantly higher specificity (98%) in contrast to sensitivity (46.2%) [25].

The literature shows that many other researchers have further explored the utility of urine strips. For example, Huy et al. found sensitivity and specificity of 91% and 95%, respectively, in detecting leukocytes in CSF in 200 patients with suspected meningitis [15].

Similarly, another study of 150 patients with suspected meningitis by Heckmann et al. revealed 92.6% sensitivity and 100% specificity for leukocyte detection and 77.8% sensitivity and 100 % specificity for protein detection in CSF [26]. If we compare these results with the results shown in studies by Mazumder et al. [21] and Gupta et al. [20], it adds further validation to the accuracy of urine reagent strips.

Moosa et al. did a similar study in 1995 and they found 97% sensitivity and 100% specificity [27]. This again is very promising and favorable evidence for the use of these urine strips in relevant clinical areas.

Manthappa et al. did another study on the role of leukocyte esterase in diagnosing meningitis, and the sensitivity for leukocyte esterase ++ and above, with a cut-off value of 100 cell/mm^3^ on microscopy, revealed a sensitivity of 100% and specificity of 98%. Negative and positive predictive values were also measured, indicating values of 100% and 90%, respectively [28].

Molyneux did a literature review and found urinary reagent strips helpful in diagnosing meningitis where laboratory infrastructure is not well equipped or where there are no labs available [29].

While all the above studies point out the potential use and accuracy of urinary reagent strips and their use in resource-lacking settings, it is also important to look for the limitations. This study had several limitations, such as not looking for bacteria, tuberculous, and sterile meningitis and missing other cases with comorbid conditions. The variable results among various studies are the main limiting factor for its use in a larger population. Additionally, the training of health professionals for the use of these strips, especially in healthcare systems with poor infrastructure, is another challenge. Importantly, this study was done in a resource-lacking setup, posing a challenge to its wide application in advanced well-equipped infrastructures. Similarly, this study shows the detection of leukocytes in the CSF, but it was not able to differentiate between neutrophils and lymphocytes, which is another limitation. This needs multi-center data to validate its wide applicability.

Nevertheless, the study also highlighted some advantages, as the test showed reasonable sensitivity and specificity, and was relatively cheap compared with other CSF chemistries.

## Conclusions

CSF pleocytosis is detectable with urine reagent strips, which, if negative, could exclude bacterial meningitis in places without laboratory infrastructure. The diagnostic accuracy, sensitivity, and specificity of the urine strip in our study was 92.52%, with a sensitivity of 90.62% and specificity of 93.98%, which can be used in resource-restrained settings. The urine strip method is a quite quick and cost-effective method. 

However, it is worth mentioning that the variability in the results of available studies suggests that more detailed standardization and collaboration are needed and that defined levels of proteins and CSF leukocytes may improve the results and support its wide application.
